# Pharmacometabolic Effects of Pteryxin and Valproate on Pentylenetetrazole-Induced Seizures in Zebrafish Reveal Vagus Nerve Stimulation

**DOI:** 10.3390/cells12111540

**Published:** 2023-06-04

**Authors:** Adrianna Skiba, Daniele Pellegata, Veronika Morozova, Ewelina Kozioł, Barbara Budzyńska, Simon Ming-Yuen Lee, Jürg Gertsch, Krystyna Skalicka-Woźniak

**Affiliations:** 1 Department of Chemistry of Natural Products, Medical University of Lublin, 20-093 Lublin, Poland; 2 Institute of Biochemistry and Molecular Medicine, University of Bern, 3012 Bern, Switzerlandveronika.morozova@unibe.ch (V.M.); 3 Independent Laboratory of Behavioral Studies, Medical University of Lublin, 20-093 Lublin, Poland; barbarabudzynska@umlub.pl; 4 State Key Laboratory of Quality Research in Chinese Medicine, Institute of Chinese Medical Sciences, University of Macau, Macao 519020, China; simonlee@umac.mo

**Keywords:** pharmacokinetics, epilepsy, LC-MS analytics, valproate, amino acids, metabolomics, vagus nerve, autonomous nervous system

## Abstract

Zebrafish (Danio rerio) assays provide a versatile pharmacological platform to test compounds on a wide range of behaviors in a whole organism. A major challenge lies in the lack of knowledge about the bioavailability and pharmacodynamic effects of bioactive compounds in this model organism. Here, we employed a combined methodology of LC-ESI-MS/MS analytics and targeted metabolomics with behavioral experiments to evaluate the anticonvulsant and potentially toxic effects of the angular dihydropyranocoumarin pteryxin (PTX) in comparison to the antiepileptic drug sodium valproate (VPN) in zebrafish larvae. PTX occurs in different Apiaceae plants traditionally used in Europe to treat epilepsy but has not been investigated so far. To compare potency and efficacy, the uptake of PTX and VPN into zebrafish larvae was quantified as larvae whole-body concentrations together with amino acids and neurotransmitters as proxy pharmacodynamic readout. The convulsant agent pentylenetetrazole (PTZ) acutely reduced the levels of most metabolites, including acetylcholine and serotonin. Conversely, PTX strongly reduced neutral essential amino acids in a LAT1 (SLCA5)-independent manner, but, similarly to VPN specifically increased the levels of serotonin, acetylcholine, and choline, but also ethanolamine. PTX dose and time-dependent manner inhibited PTZ-induced seizure-like movements resulting in a ~70% efficacy after 1 h at 20 µM (the equivalent of 4.28 ± 0.28 µg/g in larvae whole-body). VPN treated for 1 h with 5 mM (the equivalent of 18.17 ± 0.40 µg/g in larvae whole-body) showed a ~80% efficacy. Unexpectedly, PTX (1–20 µM) showed significantly higher bioavailability than VPN (0.1–5 mM) in immersed zebrafish larvae, possibly because VPN in the medium dissociated partially to the readily bioavailable valproic acid. The anticonvulsive effect of PTX was confirmed by local field potential (LFP) recordings. Noteworthy, both substances specifically increased and restored whole-body acetylcholine, choline, and serotonin levels in control and PTZ-treated zebrafish larvae, indicative of vagus nerve stimulation (VNS), which is an adjunctive therapeutic strategy to treat refractory epilepsy in humans. Our study demonstrates the utility of targeted metabolomics in zebrafish assays and shows that VPN and PTX pharmacologically act on the autonomous nervous system by activating parasympathetic neurotransmitters.

## 1. Introduction

Zebrafish assays provide several well-established experimental in vivo models in different biological areas, such as embryology, pharmacology, and toxicology [1]. Zebrafish larvae are suitable to pharmacologically profile chemicals in the contexts of chemical safety, drug discovery, and environmental risk assessment [2]. The sufficiently high homology to mammalian morphology and biology also makes zebrafish an attractive animal model to study neurological disorders, including epilepsy [3,4,5]. Moreover, zebrafish larvae exhibit a wide range of complex behaviors and thus offer behavioral models for different paradigms [6]. Thus, in a first-line pharmacological screening, zebrafish assays can be complementary to mammalian models of different central nervous system (CNS) diseases [7,8], allowing for large-scale throughput profiling of biologically active substances.

A major challenge in the application of zebrafish assays, with respect to correctly interpreting data, lies in the lack of knowledge about the bioavailability of pharmacologically active substances. Water-soluble chemicals can be added directly to the aquarium to enable uptake by the mouth, skin, and gills [9]. As the general route of administration is the addition of test compounds into the zebrafish larvae medium, the whole-body bioavailability upon immersion often remains unknown. This problem is particularly intrinsic to the diverse biophysical properties of natural products which range from charged polar and amphiphilic to apolar that exhibit poor solubility in aqueous solutions. To date, relatively few studies have measured zebrafish whole-body uptake of a particular drug upon immersion at its bioactive concentration [2,10,11,12,13].

**Figure 1:**
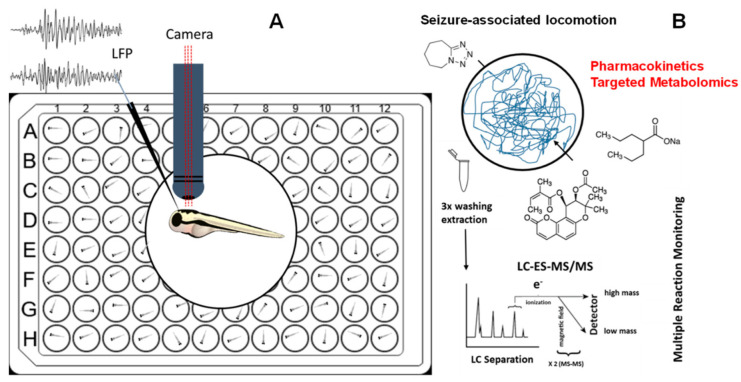
(**A**) 6-dpf zebrafish larvae were incubated in a medium containing vehicle control (DMSO), different concentrations of valproic acid (VPN), and pteryxin (PTX) for 1 h and 18 h prior to seizure induction by pentylenetetrazole (PTZ). For all experiments locomotion was measured and for PTX (20 µM) also local field potential (LFP) recordings were acquired. (**B**) Pharmacometabolomic effects of VPN and PTX alone or in combination with PTZ in washed zebrafish larvae whole-body were measured quantitatively by LC-ESI-MS/MS using multiple reaction monitoring (MRM).

**Figure 2:**
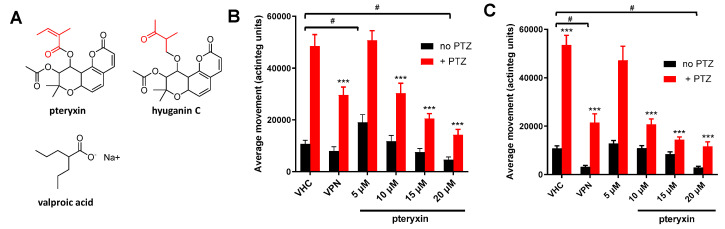
(**A**) Chemical structures of pteryxin, hyuganin C, and valproic acid (VPN). The distinct acyl substitutions are indicated in red. (**B**) Anticonvulsant activity of different concentrations of pteryxin and positive control valproate VPN (5 mM) after 1 h immersion prior to PTZ treatment. Y-axis represents the average movements of larvae measured in actinteg units. (**C**) Anticonvulsant activity of different concentrations of pteryxin and positive control (VPN) (5 mM) after 18 h immersion prior to PTZ treatment. Data show mean values ± SD of at least three independent experiments, n = 10. The Post hoc Tukey’s test indicates significant differences in the PTZ-treated group (red bars) between pteryxin and vehicle (VHC) treated groups, *** *p* < 0.001. In the no PTZ group (black bars), VPN and PTX time-dependently altered average movements at low and high concentrations compared to VHC, # *p* < 0.05.

To gain a better understanding of drug action in vivo, and to make zebrafish-based pharmacological experiments more comparable, whole-body concentration measurements using high-end analytical methodologies, such as liquid chromatography-electrospray ionization mass spectrometry (LC-ESI-MS/MS), provide tools to combine pharmacology and biochemistry (Figure 1). In this study, we compared the bioavailability and the anticonvulsant effects of pteryxin (PTX) (Figure 2A), an angular dihydropyranocoumarin, with sodium valproate (VPN) (Figure 2A) in a validated zebrafish model of seizure-like behaviour induced by pentylenetetrazole (PTZ) [14]. PTX is a structural analogue of the previously tested hyuganin C [15]. The anticonvulsant effects of PTX were measured as a reduction of seizure-like movements and subsequently confirmed by local field potential (LFP) measurements. The epilepsy-like seizures in zebrafish can mimic forms of epilepsy in mammals and thus provide a relatively straightforward first readout in profiling anticonvulsant agents [16]. This is corroborated by the fact that PTZ-induced seizures in zebrafish are inhibited by major anti-epileptic drugs such as VPA (valproic acid), but also diverse experimental substances that show comparable anticonvulsant effects in both mice and zebrafish models, including coumarins [16,17].

As will be discussed below, there is a possible association between coumarins found in Apiaceae and the traditional use of antiepileptic botanical drugs. In Europe, ancient traditional remedies for epilepsy are documented by nine Renaissance herbal books mentioning species from the genera *Angelica*, *Heracleum*, *Peucedanum*, *Seseli*, *Ferula* and *Eryngium* [18]. However, the reverse translation of this anecdotal knowledge and pharmacological validation in vivo, in the sense of corroboration or refutation, is challenging. The furanocoumarins imperatorin and xanthotoxin, as well as the simple coumarin osthole, have been shown to exert protective effects in maximal electroshock seizure in mice at relatively high doses [19]. Linear furanocoumarins, such as lucidafuranocoumarin A and bergamottin, have shown protective effects against PTZ-induced seizures in zebrafish assays [20]. However, there is limited information regarding the possible anticonvulsive effects of pyranocoumarins, which are major constituents of antiepileptic traditional botanical drugs. In our previous study, hyuganin C (Figure 2A), a structural analogue of PTX, showed protective effects against PTZ-evoked seizures in a locomotor assay, which were confirmed by Power Spectral Density (PSD) analysis of electrophysiological experiments [17].

Valproic acid (VPA) is an anticonvulsant drug indicated for the treatment of epilepsy and is occasionally used in the treatment of bipolar disorder [21,22]. The mode of action of VPA remains unclear, though proposed mechanisms include modulation of GABA levels, blocking of voltage-gated sodium channels, and inhibiting histone deacetylases [23,24]. VPA is clinically available in several forms including VPA alone, sodium valproate (VPN), and VPN in combination with VPA [21]. For in vitro and in vivo experiments, possible pharmacokinetic differences between these forms are not unexpected as the VPN salt needs to dissociate to release the cell-permeable VPA. In the PTZ zebrafish model, both VPN and VPA have been shown to exert protective effects using high micromolar concentrations [14]. VPA also induces significant toxic effects in zebrafish embryos and larvae, both acutely and upon prolonged administration [25]. While VPN is entirely orally bioavailable (~90%) in mammals [26,27], the bioavailability of VPN in immersed zebrafish is unknown, especially with respect to the ionization constant of VPN in zebrafish medium. Since different concentrations and immersion times have been used, whole-body uptake measurements of compounds, upon thorough washing, provided important information about the actual effective concentrations in the organism. In this study, we measured larvae whole-body metabolites upon immersion with PTZ, PTX, and VPN, respectively, by targeted metabolomics harnessing LC-ESI-MS/MS. This methodology enabled a first interrogation on common underlying modes of action of PTX and VPN in the PTZ epilepsy-like zebrafish model by comparing their pharmacometabolomic effects. Based on data from targeted metabolomics, differential, and comparable effects of VPN and PTX on seizure-associated locomotion and their whole-body absorption could be obtained (Figure 1). A major finding of this study is that both compounds appear to target similar metabolic pathways, leading to a pronounced rise in acetylcholine (ACh) and serotonin (5-HT) levels, strongly indicative of vagus nerve activation.

## 2. Materials and Methods

### 2.1. Zebrafish

Zebrafish (Danio rerio) stocks of the AB strain were maintained at 28.5 °C, on a 14/10 h light/dark cycle under standard aquaculture conditions, and fertilized eggs were collected via natural spawning. Embryos were reared under 24 h light conditions in embryo medium: 1.5 mM HEPES, pH 7.1–7.3, 17.4 mM NaCl, 0.21 mM KCl, 0.12 mM MgSO4, and 0.18 mM Ca(NO_3_)_2_ at 28.5 °C. For immersion 6 days post-fertilization (dpf) and for all measurements, 7-dpf larvae were used. The zebrafish experiments described here were approved by the Local Ethics Committee in Lublin (license no: 109/2018).

### 2.2. Animal Treatment

PTX (>95% by HPLC) was obtained as described previously [27]. All compounds were dissolved in dimethyl sulfoxide (DMSO) and diluted in an embryo medium to achieve a final DMSO concentration of 1% *w/v*. Embryo medium prepared with DMSO to a final concentration of 1% *w/v* served as vehicle control (VHC). PTZ (Sigma-Aldrich, Poznań, Poland) was dissolved at a concentration of 40 mM in the embryo medium. The control anticonvulsant agent used in this study was VPN (Sigma-Aldrich, Poznań, Poland).

### 2.3. Toxicological Evaluation

The toxicity of compounds and extracts was evaluated by determining the maximum tolerated concentration (MTC), defined as the maximum concentration that did not cause death and where not more than 2 out of 10 larvae exhibited any signs of locomotor impairment including lack of touch response after an 18 h immersion period [13]. Each larva was checked under the microscope for signs of acute locomotor impairment: weak response upon the light touch of the tail with a fine needle [28,29], loss of posture, body deformation, bulging of the eyes out of their sockets, slow or absent heartbeat, and death. Zebrafish larvae were incubated with different concentrations of extracts and tested compounds. Immersions were performed at 28.5 °C in complete darkness for 18 h. PTX was tested at a concentration range of 25–100 µM.

### 2.4. Locomotor Tracking

The 6-dpf larvae were preincubated in 100 µL of 1% DMSO, VPN (5 µM) [13] or tested compounds, for 1 and 18 h in individual wells of a 96-well plate at 28.5 °C in the dark. 100 µL of embryo medium or 100 µL of a 40 mM PTZ solution was added to obtain a final concentration of 20 mM of PTZ in each well [30]. Larvae were habituated for 5 min in a dark chamber of an automated tracking device (ZebraBox system; Viewpoint, Lyon, France). Then, the locomotor activity without proconvulsant treatment was measured in the dark. The total locomotor activity was quantified using ZebraLab software (Viewpoint, Lyon, France) and was expressed in “actinteg”’ units, which is the sum of all pixel changes detected during the period defined for the experiment [30]. After the evaluation of preincubated larvae movements, PTZ solution was added, and the locomotor behavior was measured for 30 min for each condition. All tracking experiments were performed at least in triplicate.

### 2.5. Local Field Potential

The 6-dpf larvae were incubated with 100 μL of 1% DMSO and tested compounds for 18 h in individual wells of a 96-well plate at 28.5 °C in the dark. Non-invasive local field potential (LFP) was recorded from the optic tectum of zebrafish as described before [11,31]. Before the recording, the treated larvae were immersed for 15 min in 100 µL of VHC or 20 mM PTZ. After immersion, a larva was immobilized in 2% low melting agarose (Invitogen, Waltham, MA, USA) and the signal electrode (Hilgenberg, Germany) was placed on the surface of the head above the optic tectum. The differential signal between the recording electrode and the reference electrode was amplified 10,000 times by EXT-02F/2 extracellular amplifier (NPI Electronic, Tamm, Germany), band pass filtered at 3–300 Hz and digitized at 2 kHz via a PCI-6251 interface (National Instruments, Austin, Texas, USA) with WinEDR (John Dempster, University of Strathclyde, Glasgow, Scotland). A HumBug noise eliminator (Quest Scientific, Hofheim, Germany) was used to remove 50–60 Hz noise. The recordings and the quantification of the signal from epileptiform brain discharges by Welch’s power spectral density (PSD) analysis were performed as described before [32,33,34].

### 2.6. LC-MS/MS Quantification and Estimation of Bioavailability of VPN (as VPA) and PTX in Larvae

To evaluate concentrations of the tested compounds and amino acid content in zebrafish, larvae were incubated at 28.5 °C in the dark in six well plates for 5 min, 30 min, 1 h, and 18 h, respectively, with 5 mL of VPN and PTX at the active concentration determined in the PTZ epilepsy-like zebrafish model. After immersion larvae were plated in 1% BSA for 15 s and washed thoroughly by shaking to remove all compounds from the larvae surface. Larvae were euthanized following regulatory guidelines (EU Directive 2010/62/EU) [35] and then transferred to Eppendorf tubes, where an excess of liquid was removed, and samples were put into a freezer at −80 °C. For quantification, six larvae were pooled before homogenization. Three experiments were carried out in independent days.

#### 2.6.1. Sample Extraction

The 200 µL of iced cold Sterile PBS was added to the samples (besides samples containing PTX 20 µM in the medium). These were homogenized with the Ultrasonic Processor UP50H (Hielscher): 1 cycle—60% amplitude. The run consisted of two cycles of 15 s with a rest of 50 s in ice for each sample. The samples for assessing the bioavailability of VNA and PTX and amino acids and neurotransmitters quantification were prepared the same way. To 100 µL of homogenate 200 µL of the internal standard (IS) Rolipram (Sigma Aldrich, Buchs, Switzerland) (20 ng/mL) for the positive MS experiment, and VPA d-6 (Sigma Aldrich, Buchs, Switzerland) (50 µg/mL) for the negative MS experiment were added. Samples were vortexed for 10 s and then centrifuged at 12,000 rpm for 10 min at 4 °C. After centrifugation, 150 µL of supernatant was transferred into LC-MS vials already containing 120 µL of MilliQ water. Then, 10 µL was injected into the LC-MS/MS system.

#### 2.6.2. LC-ESI-MS/MS Measurements

A hybrid triple quadrupole 4000 QTRAP mass spectrometer (AB Sciex Concord, ON, Canada) was used with a Shimadzu UFLC (Shimadzu Corporation, Kyoto, Japan) with a cooled auto-sampler. The sample temperature was maintained at 4 °C in the auto-sampler prior to analysis. The LC column was Synergi 4 µm Fusion-RP 80Å C18 50 × 2 mm, Phenomenex, maintained at 45 °C. The system was operated first in positive and after in negative mode with the same elution mobile phases and gradient method. Mobile phases were as follows: water with 0.1% formic acid (solvent A) and methanol with 0.1% formic acid (solvent B). The gradient started at 5% B, hold for 0.2 min, then it was increased linearly to 95% B at 2.5 min, kept until 4.2 min, with subsequent re-equilibration at 5% B until 5.0 min. The flow rate was 0.80 mL/min. Peaks were integrated, and the analyst software version 1.6.2 (AB Sciex Concord, ON, Canada) was used for quantification. Identification of compounds in samples was confirmed by comparison of precursor and product ion m/z values and LC retention times with standards. The following Multiple Reaction Monitoring (MRM) transitions were selected for quantification of the analytes: Positive mode: PTX m/z 409.2→175.2, (IS: Rolipram 276.1→208.2). Negative mode: Valproic acid m/z 143→143, (IS: Valproic Acid-d6 149→149). Calibration curves for the biological matrix were generated by plotting the peak area ratio (y) of VPN/VPA and PTX to IS versus its nominal concentration (x) using a weighted (1/x^2^) linear regression model. An appropriate coefficient of correlation (R^2^ ≥ 0.9900) was obtained for both compounds (Figure S1).

### 2.7. Amino Acids and Neurotransmitters Quantification

#### 2.7.1. Sample Preparation and Extraction

The samples were prepared as described before. To 80 µL of the homogenate 320 µL of MeOH was added to precipitate proteins at a volume dilution (1:4). After centrifugation at max speed (20,000 rpm) at 4 °C for 10 min, 50 µL were diluted with 200 µL of 1:1 MeOH:1 mM EDTA in Water (Dil 1:4). Samples were centrifuged at max speed at 4 °C for 10 min, and 50 µL were taken into the LC-MS insert vials, 5 µL of the internal standard mix (ethanolamine_d4, dopamine_d4, methionine_d3, serine_d3, lysine_d4 glycine_d5, and acetylcholine_d4) was added to all vials. Subsequently, 10 µL was injected into the LC-MS/MS system in positive mode.

#### 2.7.2. LC-MS/MS Conditions

A hybrid triple quadrupole 4000 QTRAP mass spectrometer (AB Sciex Concord, ON, Canada) was used with a Shimadzu UFLC (Shimadzu Corporation, Kyoto, Japan) with a cooled auto-sampler. The sample temperature was maintained at 4 °C in the auto-sampler prior to analysis. The LC column used was Imtakt Intrada amino acid WAA34, 100 × 3 mm; 3 µm, maintained at 35 °C. The system was operated in positive mode. Mobile phases were: 100 mM Ammonium formate in water (mobile phase A) and 95:5:0.3 acetonitrile:water:formic acid (mobile phase B). The gradient at a flow rate of 1.00 mL/min was as follows: 0 min—92% B, 3 min—88% B, 6.4 min—70% B, 6.5–10 min—0% B, 10.1–12.9 min—92% B. The Multiple Reaction Monitoring (MRM) transitions used for the quantification of the analytes are shown in Table S1. MS peaks were integrated using the Analyst software version 1.6.2 (AB Sciex Concord, ON, Canada) for quantification. Identification of compounds in samples was confirmed by comparison of precursor and production m/z values and LC retention times with standards.

### 2.8. Statistical Analysis

All statistical analyses were performed using GraphPad Prism 9 software (GraphPad Software, Inc., San Diego, CA, USA). Values for locomotor activity were presented as mean ± standard deviation (SD). The locomotor activity of zebrafish larvae was analyzed using two-way ANOVA followed by a Bonferroni test. Values for normalized PSD data were presented as mean ± standard error of measurement (SEM). The results were analyzed using two-way ANOVA followed by post-hoc Bonferroni test and for PSD per frequency and per larva results, one-way ANOVA followed by post-hoc Dunnett test was used.

## 3. Results and Discussion

### 3.1. Determination of Anticonvulsant Activity of VPN and PTX in the PTZ Epilepsy-like Zebrafish Model

The distinctly substituted angular dihydropyranocoumarin pteryxin (PTX) (Figure 2A) was tested in a validated PTZ-induced seizure-like model using VPN as a positive control. Additionally, 20 mM of PTZ was used, as this concentration was previously shown to robustly induce convulsions (seizure-mediated locomotion) [17]. Five mM of VPN was employed as this concentration showed the most effective anti-seizure effect without apparent acute toxic effects when zebrafish larvae were immersed for 1 h.

Twenty mM PTZ significantly increased actinteg values in the measurement of locomotor activity of zebrafish larvae in all treated groups, confirming its proconvulsant effect (*p* < 0.001) (Figure 2B,C). After 1 h of pre-immersion, PTX statistically reduced the subsequent PTZ-induced convulsions (Figure 2B) (PTZ treatment [F(5, 339) = 24.75; *p* < 0.0001], VPN and different concentrations of PTX pretreatment [F(1, 339) = 199.0; *p* < 0.0001] as well as interactions between treatment and pretreatment [F(5, 339) = 8.116; *p* = 0.0001]. VPN (*p* < 0.001) and PTX at 10, 15, and 20 µM (*p* < 0.001) decreased the activity of the larvae treated with PTZ in comparison to a vehicle-PTZ-treated group. These concentrations decreased actinteg values by 37.6, 57.6, and 70.6%, respectively. Additionally, 5 µM PTX increased the average movements after 1 h, whereas 20 µM decreased locomotor activity compared to the vehicle-treated group (Figure 2B). The increased locomotor activity observed with PTX in light conditions could be indicative of effects on GABAergic or glutamatergic signaling [28].

Subsequently, we used an 18 h immersion time to mimic sub-chronic conditions. As shown in Figure 2C, longer immersion with VPN and 20 µM PTX showed significant inhibition of the locomotor activity in comparison with the vehicle-treated group, such as the acute treatment. Two-way ANOVA analysis showed a statistically significant effect in PTZ treatment [F(1, 336) = 175,4; *p* < 0.0001], 5 mM VPN and different concentrations of PTX pretreatment [F(5, 336) = 32.28; *p* < 0.0001], as well as interactions between treatment and pretreatment [F(5, 336) = 17.25; *p* = 0.0001]. Both 5 mM VPN (*p* < 0.001) and PTX at 10, 15, and 20 µM (*p* < 0.001) decreased the activity of PTZ-treated larvae in comparison with a vehicle-PTZ-treated group. The concentrations 10, 15 and 20 µM decreased locomotor activity by 61.4%, 73.3%, and 78.2%, respectively (Figure 2C).

**Figure 3:**
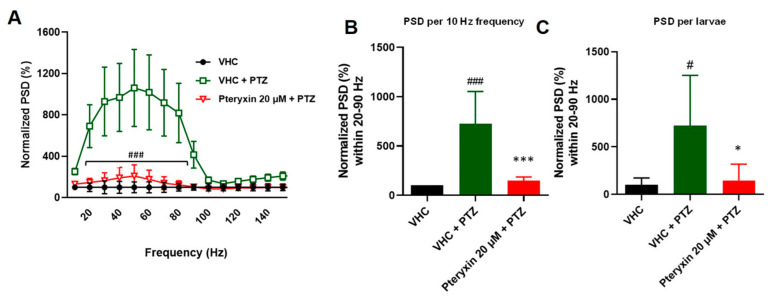
Electrophysiological measurements of anticonvulsant activity using power spectral density (PSD) analysis of 20 µM pteryxin treatment (18 h) in the PTZ-induced seizure model. (**A**) PSD of the local field potentials (LFPs) in the range of 0–150 Hz were measured. Raw data were normalized to vehicle control (VHC—1% DMSO in E3 medium). Outliers were identified by the ROUT test (Q = 1%). The post hoc Bonferroni’s test from at least nine independent experiments showed significant differences (20–90 Hz) between pteryxin treatment (red line) and vehicle (VHC) and PTZ treatment (green line), ### *p* < 0.05. (**B**) Normalized data were plotted as mean values (SEM) PSD per 10 Hz. The post hoc Tukey’s test indicated a significant difference between VHC (black bar) and PTZ treatment (green bar), ### *p* < 0.0001 and pteryxin treatment (red bar) versus VHC treatment (green bar), *** *p* < 0.05. (**C**) When PSD per 10 Hz and per larva over the 0–150 Hz region were analyzed, the post hoc Tukey’s test indicated a significant difference between VHC and PTZ, # *p* < 0.05 and PTZ treatment vs. pteryxin and VHC * *p* < 0.05. Corresponding PSD analyses of the positive control VPN showing comparable effects have been published previously [36].

To confirm the results of the locomotor test, LFP recordings were performed to measure epileptiform brain discharges induced by PTZ. This method allows for the detection of electrographic activity in the zebrafish brain and is analogous to an electroencephalogram (EEG) performed in humans. LFP recordings were examined by a power spectral density (PSD) analysis by computing average power in consecutive 10 Hz frequency bands ranging from 1–160 Hz and normalized against VHC-treated larvae. As shown in Figure 3A, after 18 h immersion, PTX showed inhibitory effect on the PTZ-induced epileptiform activity, as there was a significantly lower PSD within the frequency range between 10–50 Hz (frequency F(2, 27) = 6.623, *p* = 0.0046, treatment (14, 378) = 6.825, *p* < 0.0001 and interaction F(28, 378) = 5.714, *p* < 0.0001). Similarly, when PSDs were plotted as mean PSD per condition over the 1–50 Hz region (Figure 2A), there was a significant decrease in the PSD values by 99.8% in comparison to PTX treated group. Qualitatively similar effects on PSD are typically observed with VPN [36]. Given the novelty of the PTX-mediated anticonvulsive effects, we only measured PTX in LFP recordings.

In a recent study conducted by our group [17], similar results were observed for hyuganin C, a structural analogue of PTX. Hyuganin C showed protective properties by decreasing the seizure-like behavior of PTZ-treated larvae by 27% and 35% at 10 µM and 20 µM, respectively, in comparison with the vehicle-PTZ-treated group. In the electrophysiological study, hyuganin C decreased the normalized PSD by 65%. Although pteryxin and hyuganin C are structural analogues, the substitution at the C-10 atom in pteryxin in the molecular scaffold increased efficiency. Although structure-activity relationships were not the topic of this study, we previously reported that pimpinellin, an angular furanocoumarin, also showed anticonvulsive effects [17]. Pimpinellin decreased seizure-like behavior in a concentration-dependent manner, with efficacy ranging from 25 to 60% reduction, and decreased normalized PSD by 81%. This suggests that angular coumarins may generally show anticonvulsive effects in this model and that the substitutions further increase their potency and efficacy.

### 3.2. Measurment of Compound Concentration in Larvae Using LC-MS/MS Quantification

**Figure 4:**
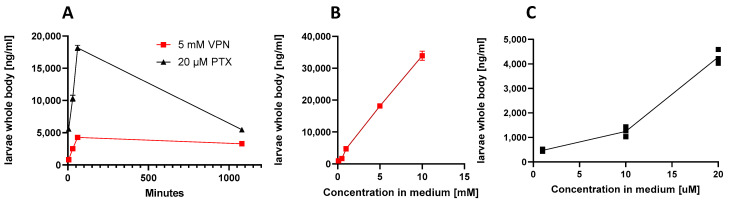
Whole-body uptake of VPN and pteryxin (PTX) upon zebrafish larvae immersion. (**A**) Kinetics of uptake with a fixed anticonvulsive concentration of VPN (5 mM) and PTX (20 μM). (**B**) Concentration-dependent increase of larvae whole-body uptake of VPN. (**C**) Concentration-dependent uptake of larvae whole-body uptake of PTX. Experiments show mean values of at least three independent experiments, n = 6. In some cases, more experiments were performed and are shown as additional dots. Data are also shown in Table 1.

To associate compound absorption with potency, we measured whole-body uptake kinetics of VPN/VPA and PTX, respectively, upon immersion with different concentrations. To that aim, we generated MRM methods using LC-MS/MS for the quantification of VPA and PTX (see methods). Since both VPN and VPA are detected as VPA fragmentation ion peaks in the matrix they cannot be distinguished. VPN/VPA dose and time-dependently (5 min, 30 min, 1 h) increased in whole-body larvae, showing a nonlinear dose–response relationship and a linear time-dependency (Figure 4A). After 18 h the level detected in the body was significantly decreased, indicative of VPA detoxification/metabolism. As shown in Table 1, VPN was very poorly absorbed by zebrafish larvae, which, given its outstanding oral bioavailability in mammals [27], was surprising. Though VPN dissolves in water due to electrical charges and because both water and salt compounds are polar, the ionization constant and dissociation kinetics may depend on different factors, such as medium composition and temperature (28.5 °C in zebrafish medium versus 37 °C in mammals). When calculated as % absorbed, the lowest concentration of 0.1 mM showed the highest bioavailability (~6%) which may be explained by the salt: solvent ratio favoring dissolution of ions. Within a 1 h range, VPN (5 mM) showed linear absorption kinetics (5 min—0.77%, 30 min—1.44%, 1 h—2.52%) (Figure 4B). VPN has been extensively studied in zebrafish for both anticonvulsive and toxic effects [25,29]. Given that VPA is less soluble in aqueous solutions (1.2 mg/mL), VPN is preferentially used over VPA in zebrafish work. Despite the numerous toxic and pharmacological effects of this drug [14,25], we could not find data on its bioavailability in zebrafish. Noteworthy, at 5–10 mM of VPN, similar anticonvulsive concentrations were reached in zebrafish larvae (estimated 235.3 ± 10.1 µM at 10 mM) as reported from human plasma [30], thus justifying the relatively high doses (mM) generally employed in this assay. Since mass spectrometry cannot differentiate between VPN and VPA, the best explanation for the observed poor absorption of VPN is a slow dissociation of VPA from sodium salt in the medium. VPN is not cell permeable, unlike the branched-chain saturated fatty acid VPA. It is known that upon prolonged immersion (72 h), VPA induces significant neurotoxic effects in zebrafish [25] and already µM concentrations seem to induce craniofacial malformations via disturbed cranial neural crest cell function leading to defects in cartilage and bone formation. The toxicological difference in potency between VPA and VPN confirms our hypothesis that VPN poorly dissociates from VPA which is the bioavailable form. Such toxicity is not noticeable after short treatment [31]. We have seen potentially toxic effects, i.e., a decrease in locomotion, upon immersion with VPN for 18 h (Figure 2B).

As shown in Table 1, PTX was efficiently absorbed by zebrafish larvae. The uptake was time- and dose-dependent (Figure 4A,C). PTX amounts increased with increasing immersion time and concentration in the medium, reaching 55% at 20 μM after 1 h immersion. In contrast to VPA, after 18 h, the concentration of PTX in larvae was not strongly decreased (Figure 4A), suggesting that this coumarin was metabolically stable in zebrafish larvae. The bioavailability of PTX was previously studied in mice upon oral administration of 100 mg/kg. PTX was detected in mouse plasma at 15 min after oral administration with a C_max_ at 120 min (976.04 ng/mL). The estimated t_1/2_ for PTX was 1.46 h [32]. The rapid and efficient (50%) whole-body uptake in zebrafish stays in line with the data from mice. PTX was easily absorbed by zebrafish larvae and, as the blood-brain barrier is not fully developed at stage of 7-dpf zebrafish, this may indicate that most of the absorbed compound may reach the brain and directly impacts the CNS [33,34].

Our data show that the amount of PTX in larvae whole-body after 1 h and 18 h of immersion do not exactly correspond with the anticonvulsive effects, which were slightly more efficient upon prolonged treatment (18 h) compared to acute treatment (1 h) (Figure 2B and Figure 4A). Thus, we cannot exclude that PTX metabolizes to even more active metabolites or that 1 h treatment results in prolonged effects at 18 h. Although the standard testing time in the PTZ epilepsy-like zebrafish model is generally 18 h pre-immersion of zebrafish larvae with test compounds, according to our data, the immersion period can be decreased to 1 h (Figure 2B), allowing for better differentiation between pharmacokinetic data and pharmacological effects of acute and sub-chronic administration, respectively.

### 3.3. Pharmacometabolic Changes on Amino Acid and Neurotransmitter Levels in Larvae Measured by LC-MS/MS

**Figure 5:**
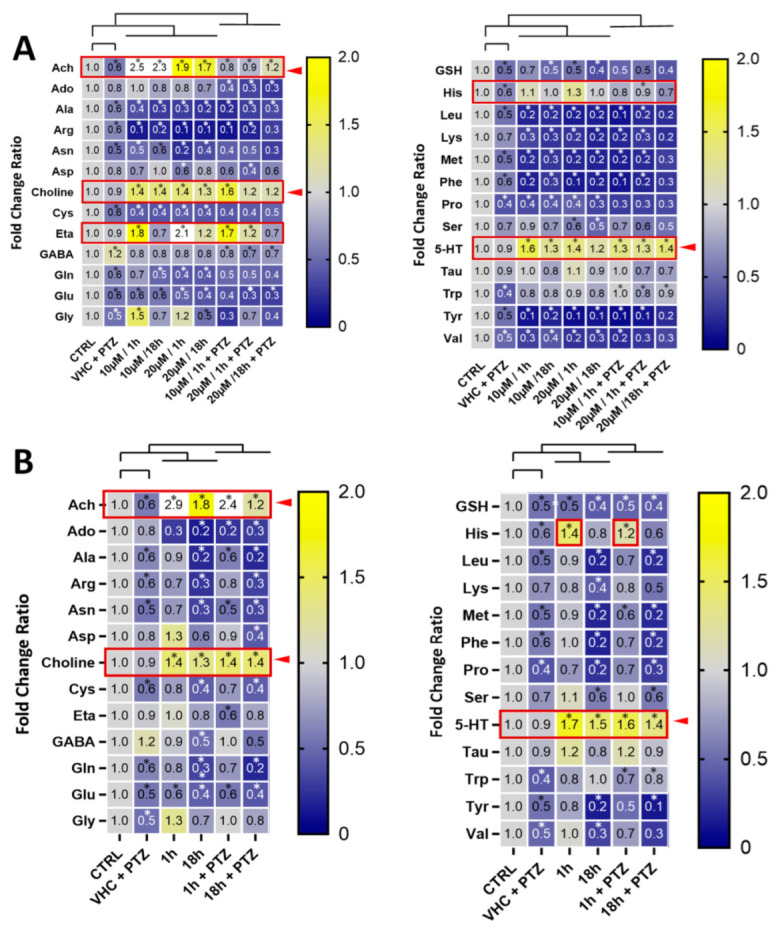
Pharmacometabolic effects of compounds in zebrafish larvae and signature of vagus nerve stimulation. Heatmap of fold changes of AA and neurotransmitter levels in comparison to vehicle (VHC)-treated control in zebrafish larvae whole-body after treatment with (**A**) pteryxin after 1 h and 18 h (10 and 20 μM) with or without PTZ and (**B**) VPN after 1 and 18 h (5 mM) with or without PTZ. Data were derived from three independent experiments, n = 6. Significant differences by two-way ANOVA for repeated measurements with Sidak’s multiple comparisons test are indicated (* *p* < 0.05). The red boxes with arrows show compound-mediated increases in whole-body metabolite levels. Data from three independent experiments were used and six zebrafish larvae were pooled for LC-MS/MS analysis.

Amino acids (AA) play a vital role in each live organism, including protein synthesis, tissue repair, synthesis of neurotransmitters and hormones, as well as the source of energy [35]. Acute or chronic changes in AA and overall metabolite content can give insight into the possible mechanism of action of tested compounds and help better understand the pathophysiology of seizures. We developed a quantitative LC-MS/MS method (see methods) to measure AA and neurotransmitters. The low levels of catecholamines in the zebrafish samples and our lower limit of quantification (LLQ) in the method did not allow for quantification. However, acetylcholine (ACh), glutamate (Glu), serotonin (5-HT), and gamma-aminobutyric acid (GABA) could be quantified reliably. This suggests that in zebrafish whole-body larvae mainly peripheral metabolites are measured and that signals from CNS metabolites are masked from the peripheral background. This was confirmed by our attempt to measure the same metabolic signature in isolated zebrafish larvae brains which was challenging and did not generate reproducible results. Immersion with PTZ led to a decrease in the concentrations of branched-chain AA (BCAA) in all tested groups, when compared to control (Figure 5). While PTZ reduced glutamate (Glu) levels, it slightly increased GABA levels (Figure 5). It needs to be pointed out that PTZ is rather non-specific and potentially toxic and does not directly reflect the patho-biochemical processes related to forms of epilepsy observed in mammals. PTX in all tested concentrations decreased content of neutral essential AA including BCAA such as leucine (Leu) and valine (Val) (Figure 5A), yet the strongest effect was visible with 20 μM PTX after 18 h of immersion, when compared to control, decreasing some AA levels by up to 60%. Since BCAA are not synthetized in animals, only in plants, bacteria and fungi, the observed whole-body content may derive from the medium, which is rich in all AA that can be actively transported into the organism or produced by intestinal microbiota of zebrafish. BCAA are responsible for synthesis of neurotransmitters and neuromodulators, including glutamate, catecholamines, 5-HT and histamine. In mammals, both BCAA and Glu concentration have been shown to be elevated during epileptic episodes [37]. Noteworthy, Leu, which was strongly reduced by PTX (Figure 5A), activates the mTOR pathway, which plays a role in development of epilepsy. The hypermodulation of the mTOR pathway is connected to seizure development, yet, the pathway in normal condition is responsible for axonal growth and regeneration [38]. Leu was also shown to enhance mTOR signalling and promoted neurodegeneration [39,40,41]. Since neutral essential AA including BCAA are transported across cell membranes by SLC7 transporters such as LAT1 (SLC7A5), and leucine (Leu) and histidine (His) levels inversely correlated (Figure 5), we speculated that PTX may modulate and act on LAT1. As shown in Figure S2, PTX strongly and specifically decreased the levels of AA typically transported by LAT1, but increased histidine. These effects were more pronounced with PTX than with VPN (Figures S2 and S3). In a follow-up experiment, PTX did not show any effect on the LAT1-mediated leucine transport in assays using LAT1 transfected cells (Figure S4). There is an overall good homology (~70%) between zebrafish and mammalian SLC transporters including LAT1 [42]. We therefore concluded that the effects of PTX on AA transported by LAT1 were indirect and rather reflected changes in peripheral metabolic processes. We noted a pronounced increase of whole-body ethanolamine (ETA) in zebrafish larvae immersed with 10 μM and 20 μM PTX for 1 h (Figure 5A). Ethanolamine is a metabolite of serine (Ser), which was, however, not strongly influenced by PTX. Ethanolamine is a precursor of phosphoethanolamine, phosphotidylcholine, choline and ACh.

Interestingly, we noticed an intriguing overall strong positive correlation (R^2^ ≥ 0.8) between ACh, choline and 5-HT levels, which were all significantly increased (Figure 5). The simultaneous increase of ACh and choline suggests that more ACh is generated/released overall, independent of ACh esterase activity which would only lead to an increase of choline. Although the administration of ACh in the CNS might have a proconvulsant effect in mammals [43], in the PTZ epilepsy-like zebrafish model we cannot differentiate between ACh levels in the brain and in the periphery. Increasing peripheral ACh levels by vagus nerve stimulation has been proposed as a potential treatment for epilepsy [44] and it is already approved by the FDA for prevention of drug-resistant seizures in humans [45].

The significant and reproducible relatively strong whole-body increase of 5-HT levels upon VPN and PTX treatment in zebrafish larvae (Figure 5) was an indication of peripheral effects. 5-HT is typically produced and released from the enterochromaffin cells in the enteric nervous system upon vagus nerve stimulation [46]. Both VPN and PTX were able to restore tryptophan (Trp) levels that were reduced by PTZ (Figure 5). Trp is the direct precursor in 5-HT biosynthesis. Noteworthy, activation of 5-HT receptors is generally associated with epilepsy treatment [47], especially the drug-resistant ones. Fenfluramine, the pharmacotherapy for drug-resistant epilepsy, is a 5-HT reupake inhibitor and 5-HT releasing agent [48,49]. Based on our data, it is possible that PTX and VPN modulate serotoninergic transmission related to the seizures induced by PTZ. VPN has previously been suggested to also act via vagus nerve stimulation in humans [50] and there is evidence that this mechanism could also be effective in zebrafish [51]. The concomitant increase of whole-body 5-HT and ACh levels observed is a typical overall peripheral metabolic signature of vagus nerve stimulation [42] which gives us the confidence to conclude that VPN and PTX exert indirect or direct stimulating effects on the vagus nerve.

## 4. Conclusions

This is the first report on the efficient anticonvulsive effects and whole-organism bioavailability of the angular dihydropyranocoumarin PTX. Similar to other coumarins [17], PTX showed a dose-dependent protective effect of PTZ-induced seizure-like movements that was confirmed by LFP recordings as was observed for VPN [36]. VPN, which is a commonly used positive control in this assay [36], was investigated alongside PTX to explore for the first time pharmacometabolic effects in zebrafish larvae. The observation that VPN was poorly bioavailable in zebrafish larvae was unexpected but may reflect the partial dissociation to readily bioavailable VPA in the zebrafish medium. Nevertheless, upon immersion of zebrafish larvae with 5 mM VPN in the medium, which is the concentration typically used, VPA concentrations measured in zebrafish (>100 µM) agreed with therapeutic plasma concentrations in treated humans (300–700 µM total and 30–100 µM unbound plasma valproate) [30,51]. Furthermore, LC-MS/MS analytics and targeted metabolomics in the zebrafish bioassay workflow enabled us to investigate putative modes of action of PTX and VPN that would be reflected in the targeted metabolome in the PTZ-epilepsy zebrafish model. Previous semi-quantitative and quantitative metabolic profiling studies performed in zebrafish larvae have already shown their potential in toxicopharmacology and physiology research [2,52]. Given the small tissue amounts and difficult-to-wash organs prior to analysis, the isolation of brains to measure metabolite content is challenging. Therefore, our whole-body pharmacometabolomic analyses cannot discern the origin of the metabolites, and most likely CNS changes cannot be seen because the readout rather reflects overall peripheral metabolic effects. However, this approach appears to be ideal to study the effects on the zebrafish autonomous nervous system. The typical vagus nerve stimulation metabolic signature (increased ACh from vagus and 5-HT from the enteric vagal system) observed in our study provides a feasible rationale to explain the protective effects of PTX and VPN in the PTZ epilepsy model. To our knowledge, there is no other known pharmacological mechanism that could induce such strong changes in ACh and 5-HT levels. The vagus nerve and enteric vagal innervation needed for serotonin release are well-developed in zebrafish larvae before the onset of feeding [53,54]. Emerging evidence suggests an important role for peripheral serotonin as a factor that enhances nutrient absorption and storage and influences neurological processes in the CNS [54,55]. Although further studies are needed to understand the underlying mechanism for the proposed vagus nerve stimulation by VPN and PTX in zebrafish larvae, our study illustrates that harnessing pharmacometabolomics data enables a toxicopharmacological assessment of bioavailable small molecules including natural products in this organism, and that effects on the autonomous nervous system are readily discernible. Overall, our study shows that simultaneously measuring test compound concentrations in zebrafish larvae and changes in endogenous metabolites can improve the pharmacological validity of effects observed in behavioral zebrafish assays.

## Figures and Tables

**Table 1:** Measured concentrations (mean value ± SD) of pteryxin and VPA/VPN in larvae whole-body (pooled n = 18) after 1 h and 18 h immersion quantified in three independent experiments using LC-MS/MS. Larvae were washed prior to homogenization.

Treatment/Concentration/Time	[ng/g]	[µM]	% Absorbed
Pteryxin/1 µM/1 h	468.7 ± 44.1	1.2 ± 0.1	1.2%
Pteryxin/10 µM/1 h	1243.3 ± 201.3	3.2 ± 0.5	32.2%
Pteryxin/20 µM/5 min	820.0 ± 46.3	2.1 ± 0.1	10.1%
Pteryxin/20 µM/30 min	2530.0 ± 105.4	6.5 ± 0.3	32.7%
Pteryxin/20 µM/1 h	4280.0 ± 284.8	11.1 ± 0.7	55.4%
Pteryxin/20 μM/18 h	3406.7 ± 213.6	8.8 ± 0.5	44.0%
Pteryxin/20 μM/without larvae	4090.6 ± 381.6	20.5 ± 0.1	-
VPN/VPA 100 µM 1 h	940.7 ± 40.8	6.5 ± 0.3	6.5%
VPN/VPA 500 µM 1 h	1680.0 ± 30.0	11.6 ± 0.2	2.3%
VPN/VPA 1 mM 1 h	4740.0 ± 111.4	32.9 ± 0.8	3.3%
VPN/VPA 5 mM 5 min	5596.6 ± 289.9	38.8 ± 2.0	0.8%
VPN/VPA 5 mM 30 min	10,366.7 ± 472.6	71.9 ± 3.3	1.4%
VPN/VPA 5 mM 1 h	18,166.7 ± 404.1	126.0 ± 2.8	2.5%
VPN/VPA 10 mM 1 h	33,933.3 ± 1457.2	235.3 ± 10.1	9.4%
VPN/VPA (5 mM) 18 h	5473.33 ± 225.02	37.97 ± 1.57	0.76% (metabolism)
VPN/VPA 5 mM without larvae	734,333 ± 5859	5092 ± 40.6	100%
Medium control	No detected	Not detected	-

## Data Availability

Data is contained within the article or supplementary material. Additional data presented in this study are available in [Figures S1–S4 and Table S1].

## Supplementary Materials

The following supporting information can be downloaded at: https://www.mdpi.com/article/10.3390/cells12111540/s1, Figure S1. (A) Product-ion scan spectra and proposed multiple reaction monitoring (MRM) transitions of PTX. PTX and IS (rolipram) were scanned with ESI positive and negative ion modes following analyses of standard solutions. In different ionization modes, the base peak intensity of positive ions was higher than that of negative ions. The ESI mass spectrum showed that the molecular ion [M + Na] + of PTX was at m/z 409.3. The intensity of the ion at *m/z* 409.3 was compared at fragmentor voltages of 40, 50, 80, 100, 120, and 150 V to determine the optimal collision energy. (B) Chromatograph using all MS transitions showing the internal standard (2 min) and PTX (2.3 min) in zebrafish larvae homogenate after 1 h of PTX treatment. The insert shows the gradient curve used (see methods). Figure S2. PTX reduces LAT1 substrate levels in vivo. Metabolomics of LAT1 substrates in Danio rerio quantified by LC-MS/MS. Data are shown as mean ± SD of triplicates. Statistical significance was calculated using two-way ANOVA and Dunnett’s post hoc test. * *p*-value < 0.05, ** *p*-value < 0.01, *** *p*-value < 0.001, when compared to DMSO control group; Figure S3. VPN increases LAT1 substrate levels in vivo. Metabolomics of LAT1 substrates in Danio rerio quantified by LC-MS/MS. Data are shown as mean ± SD of triplicates. Statistical significance was calculated using two-way ANOVA and Dunnett’s post hoc test. * *p*-value < 0.05, ** *p*-value < 0.01, when compared to the DMSO control group; Figure S4. [^3^H]-L-leucine uptake inhibition by PTX in MDST8-LAT1 cells. Dose–response inhibition by PTX, starting at 50 μM and inhibition by 5 μM JPH203. Preincubation with PTX for 2 h. The IC_50_ for PTX was determined using the four-parameter logistic to fit the dose-response curve in GraphPad Prism 9. Data shown as mean ± SEM of triplicate wells from three independent experiments.; Supplement Analytics, Table S1. Multiple Reaction Monitoring (MRM) transitions used [56].
