# Supplementary material for: Pharmacometabolic Effects of Pteryxin and Valproate on Pentylenetetrazole-Induced Seizures in Zebrafish Reveal Vagus Nerve Stimulation
Source: Cells. 2023 Jun 4;12(11):1540. doi: 10.3390/cells12111540 (PMC10252891; doi:10.3390/cells12111540)
Supplement: Supplementary file 1 [file cells-12-01540-s001.zip › cells-2378557-supplementary.pdf]

Pharmacometabolic effects of pteryxin and valproate on pentylenetetrazole-induced seizures in zebrafish reveal vagus nerve stimulation

Adriana Skiba[1], Daniele Pellegata[2], Veronika Morozova[2], Ewelina Koziol[1], Barbara Budzyńska[3], Simon Ming-Yuen Lee[4], Jürg Gertsch[2], Krystyna Skalicka-Woźniak[1]

[1] *Department of Chemistry of Natural Products, Medical University of Lublin, Poland*
[2] *Institute of Biochemistry and Molecular Medicine, University of Bern, Switzerland*
[3] *Independent Laboratory of Behavioral Studies, Medical University of Lublin, Poland*
[4] *State Key Laboratory of Quality Research in Chinese Medicine and Institute of Chinese Medical Sciences, University of Macau, Macao, China*

Chemicals used to quantify amino acids and neurotransmitters in zebrafish larvae samples
(*Compound name and abbreviation; Stock solution concentration and Solvent)*

All chemicals and reagents used for LC-MS/MS were obtained from Sigma Aldrich were of the purest analytical HPLC grade.

		[Stock] mg/ml	Solvent
Ach	Acetylcholine	0.01	MeOH
Dop	Dopamine	1	MeOH + 1 mM EDTA in ddH2O (1:1)
GSH	Glutathione	10	**1 mM EDTA in ddH2O (1:1)**
Leu	Leucine	2	MeOH + ddH2O (1:1)
Phe	Phenylalanine	2	MeOH + ddH2O (1:1)
Arg	Arginine	2	MeOH + ddH2O (1:1)
Lys	Lysine	2	MeOH + 1 mM EDTA in ddH2O (1:1)
Eta	Ethanolamine	2	MeOH
Ala	Alanine	2	MeOH + ddH2O (1:1)
Cys	Cysteine	4	MeOH + 1 mM EDTA in ddH2O (1:1)
Tyr	Tyrosine	2	MeOH + 1 M HCl 1 mM EDTA in ddH2O (1:1)
Ado	Adenosine	2	MeOH + 1 mM EDTA in ddH2O (1:1)
Gly	Glycine	20	MeOH + 1 M HCl 1 mM EDTA in ddH2O (1:1)
His	Histidine	1	MeOH + 1 mM EDTA in ddH2O (1:1)
Met	Methionine	2	MeOH + ddH2O (1:1)
Tau	Taurine	4	MeOH + ddH2O (1:1)
L-Dopa	3,4-dihydroxyphenilalanine	4	MeOH + 1 M HCl 1 mM EDTA in ddH2O (1:1)
GABA	4-aminobutyric acid	2	MeOH + ddH2O (1:1)
NA	Norepinephrine	2	MeOH + ddH2O (1:1)
Pro	Proline	2	MeOH + ddH2O (1:1)
Ser	Serine	1	MeOH + ddH2O (1:1)
Trp	Tryptophan	2	MeOH + ddH2O (1:1)
Asn	Asparagine (ASN)	2	MeOH + 1 M HCl 1 mM EDTA in ddH2O (1:1)
Asp	Aspartate (ASP)	2	MeOH + 1 M HCl 1 mM EDTA in ddH2O (1:1)
Choline	Choline	0.2	MeOH + 1 mM EDTA in ddH2O (1:1)
Epi	Epinephrine	2	MeOH + 1 M HCl 1 mM EDTA in ddH2O (1:1)
Glu	Glutamate (GLU)	2	MeOH + 1 M HCl 1 mM EDTA in ddH2O (1:1)
Gln	Glutamine (GLN)	2	MeOH + 1 mM EDTA in ddH2O (1:1)
Val	Valine	2	MeOH + ddH2O (1:1)
5HT	Serotonin	1	MeOH + 1 mM EDTA in ddH2O (1:1)

Selection of selected internal standards and grouping:

Ethanolamine_d4, dopamine_d4, methionine_d3, serine_d3, lysine_d4 glycine_d5 were purchased from Eurisotop France. Acetylcholine_d4 was purchased from Sigma Aldrich. The below standards were used to quantified analytes shown in brackets:

ETHANOLAMINE-D4	(ethanolamine, GABA, GSH, ADO)
DOPAMINE-D4	(dopamine, NE, Epin, L-DOPA, 5HT)
METHIONINE-D3	(MET, TRP, TYR, PHE, LEU, VAL, ALA, TAU)
SERINE-D3	(SER, GLU, GLN, ASP, ASN)
LYSINE-D4	(LYS, ARG, HIS)
GLYCINE-D5	(GLY, PRO, CYS)
Acetylcholine-D4	(Ach, Ch)

Table S1 Multiple Reaction Monitoring (MRM) transitions used for quantification of the analytes

**Analyte**	Precurson ion (m/z)	Product Ion (m/z)	Polarity
Ach	146.10	87.10	Positive
DA	154.00	137.30	Positive
5HT	177.10	160.30	Positive
LEU	132.10	86.10	Positive
LYS	147.10	84.10	Positive
PHE	166.10	120.30	Positive
GSH	308.20	179.10	Positive
ETA	62.00	44.00	Positive
ARG	175.10	69.90	Positive
CYS	122.10	76.10	Positive
VAL	118.00	72.10	Positive
ALA	90.00	44.00	Positive
TYR	182.10	136.20	Positive
ADO	268.20	136.20	Positive
GLY	76.00	30.10	Positive
HIS	156.10	110.10	Positive
MET	150.10	104.20	Positive
TAU	126.00	108.00	Positive
LDOPA	198.10	152.20	Positive
GABA	104.00	87.00	Positive
NE	152.10	107.00	Positive
PRO	116.00	70.00	Positive
SER	106.00	60.00	Positive
TRP	205.10	188.10	Positive
Ch	104.10	60.10	Positive
Epi	166.10	107.00	Positive
ASN	133.00	73.90	Positive
ASP	134.00	74.00	Positive
GLU	148.10	84.00	Positive
GLN	147.10	130.10	Positive

**Internal Standard**	Precurson ion (m/z)	Product Ion (m/z)	Polarity
Ach_d4	150.00	91.00	Positive
Ethan-d4	66.00	48.00	Positive
GLY-d5	78.00	32.10	Positive
DA-d4	158.10	141.10	Positive
Meth-d3	153.10	107.00	Positive
Lys-d4	151.20	88.00	Positive
Ser-d3	109.00	63.00	Positive

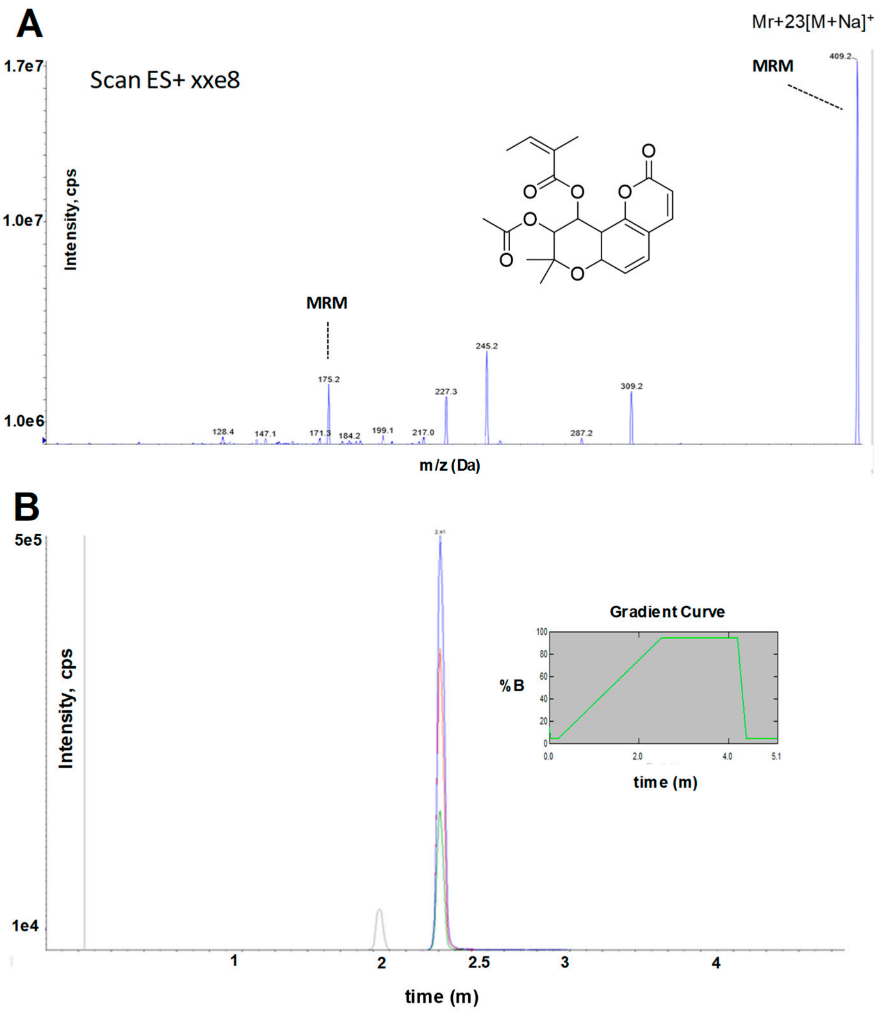

Figure S1. (A) Product-ion scan spectra and proposed multiple reaction monitoring (MRM) transitions of PTX. PTX and IS (rolipram) were scanned with ESI positive and negative ion modes following analyses of standard solutions. In different ionization modes, the base peak intensity of positive ion was higher than that of negative ion. The ESI mass spectrum showed that the molecular ion [M + Na] + of PTX was at m/z 409.3. The intensity of the ion at m/z 409.3 was compared at fragmentor voltages of 40, 50, 80, 100, 120 and 150 V to determine the optimal collision energy. **(B)** Chromatograph using all MS transitions showing the internal standard (2 min) and PTX (2.3 min) in zebrafish larvae homogenate after 1h of PTX treatment. The insert shows the gradient curve used (see methods).

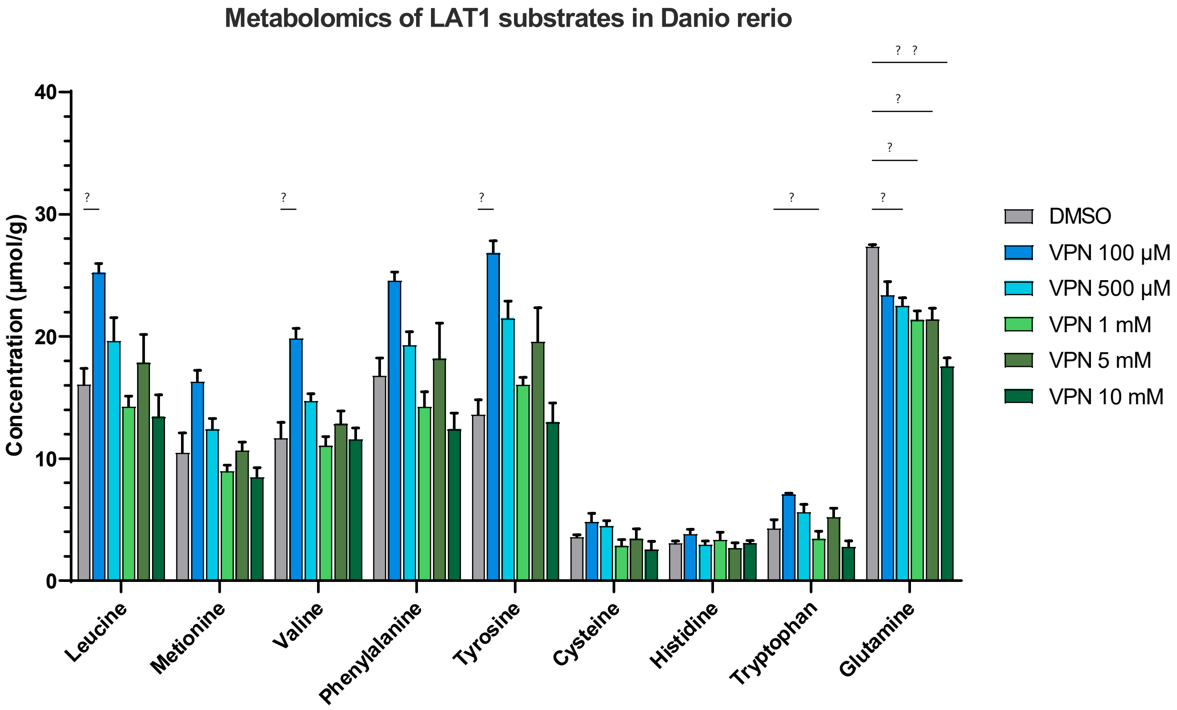

Figure S2. VPN increases LAT1 substrates levels *in vivo*. Metabolomics of LAT1 substrates in *Danio rerio* quantified by LC-MS/MS. Data are shown as mean ± SD of triplicates. Statistical significance was calculated using two-way ANOVA and Dunnett's post hoc test. * p-value <0.05, ** p-value <0.01, when compared to DMSO control group.

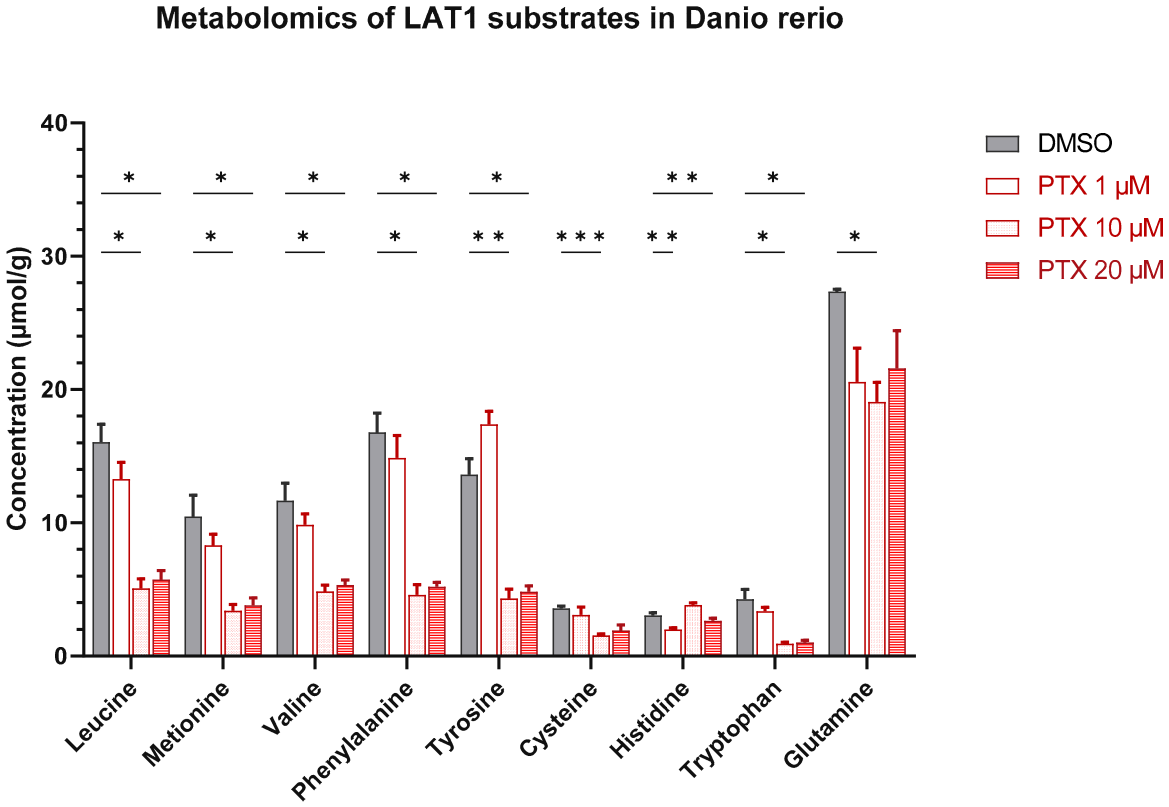

Figure S3. VPN increases LAT1 substrate levels in vivo. Metabolomics of LAT1 substrates in Danio rerio quantified by LC-MS/MS. Data are shown as mean ± SD of triplicates. Statistical significance was calculated using two-way ANOVA and Dunnett's post hoc test. * p-value < 0.05, ** p-value < 0.01, when compared to the DMSO control group.

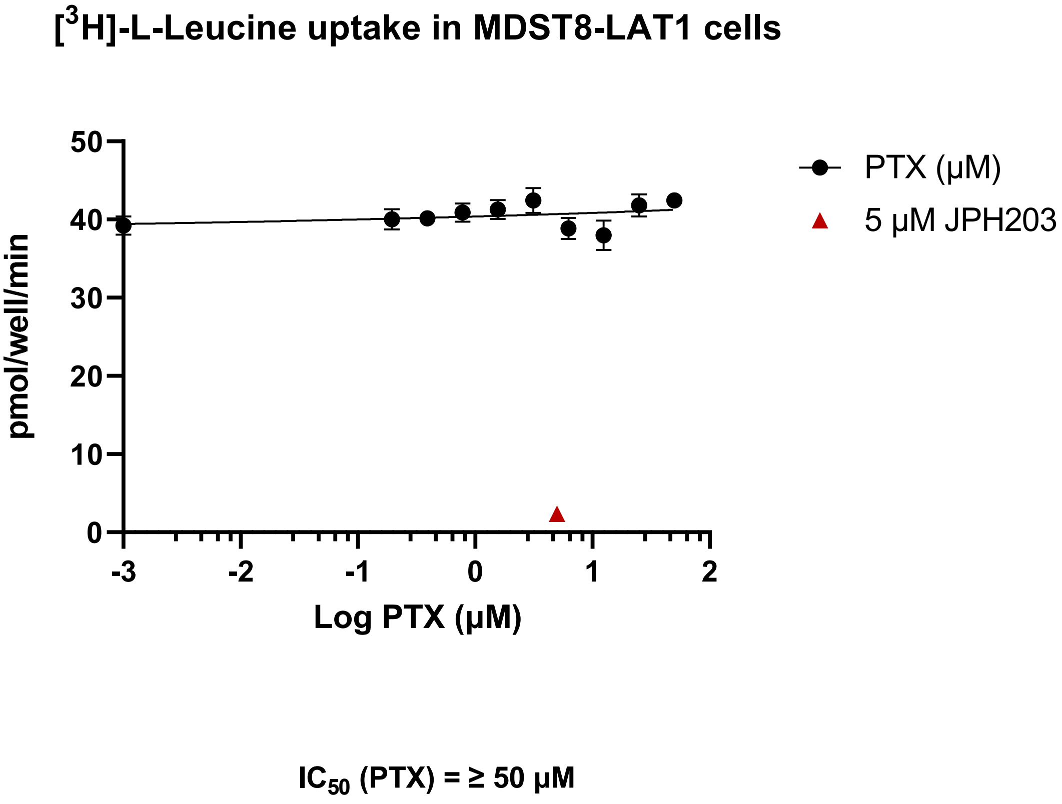

Figure S4. [3H]-L-leucine uptake inhibition by PTX in MDST8-LAT1 cells. Dose-response inhibition by PTX, starting at 50 µM and inhibition by 5 µM JPH203. Preincubation with PTX for 2 hours. The IC_{50} for PTX was determined using the four-parameter logistic to fit the dose-response curve in GraphPad Prism 9. Data shown as mean ± SEM of triplicate wells from three independent experiments.

[^{3}H]-L-Leucine uptake assay

[^{3}H]-L-Leucine uptake assay was performed as previously described [Yan *et al*., 2021]. Cells were seeded in 96-well plates with opaque bottom (Corning, 353296) at 40-60% confluency, allowing them to attach for 48 hours in a fully supplemented media. Cells were preincubated with different concentrations of PTX for 2 hours, then the cells were washed three times with pre-warmed Na^{+}-free Hank's Balanced Salt Solution (HBSS) containing 125 mM choline-Cl, 25 mM HEPES, 4.8 mM KCl, 1.2 mM $MgSO_4$, 1.2 mM KH_2PO_4, 1.3 mM $CaCl_2$ and 5.6 mM D-glucose (pH 7.4) and further incubated with the same solution for 7 minutes at 37ºC. [^{3}H]-l-Leucine uptake was assessed in the same solution containing 10 nM "hot" [^{3}H]-L-Leucine (60 Ci/mmol) (Anawa, 0140A-1) + 30 µM "cold" L-leucine. In case of 5 µM JPH203, the same procedure was followed without PTX preincubation. After 3 minutes, the solution was removed, and the cells were washed with ice-cold Na^{+}-free HBSS to terminate the uptake. Cells were lysed with scintillation solvent MicroScint-20 (Perkin-Elmer Life Sciences, 6013621) and the radioactivity was measured with a scintillation counter (TopCount NXT, Perkin-Elmer Life Sciences).

*Yan R, Li Y, Müller J, Zhang Y, Singer S, Xia L, Zhong X, Gertsch J, Altmann KH, Zhou Q. Mechanism of substrate transport and inhibition of the human LAT1-4F2hc amino acid transporter. Cell Discov. 2021 Mar 23;7(1):16. doi: 10.1038/s41421-021-00247-4. PMID: 33758168; PMCID: PMC7988154
